# Supplementary material for: Activity of botulinum neurotoxin X and its structure when shielded by a non-toxic non-hemagglutinin protein
Source: Commun Chem. 2024 Aug 13;7:179. doi: 10.1038/s42004-024-01262-8 (PMC11322297; doi:10.1038/s42004-024-01262-8)
Supplement: Supplementary file 2 — Supplementary information [file 42004_2024_1262_MOESM2_ESM.pdf]

Supplementary information

Supplementary Figure S1

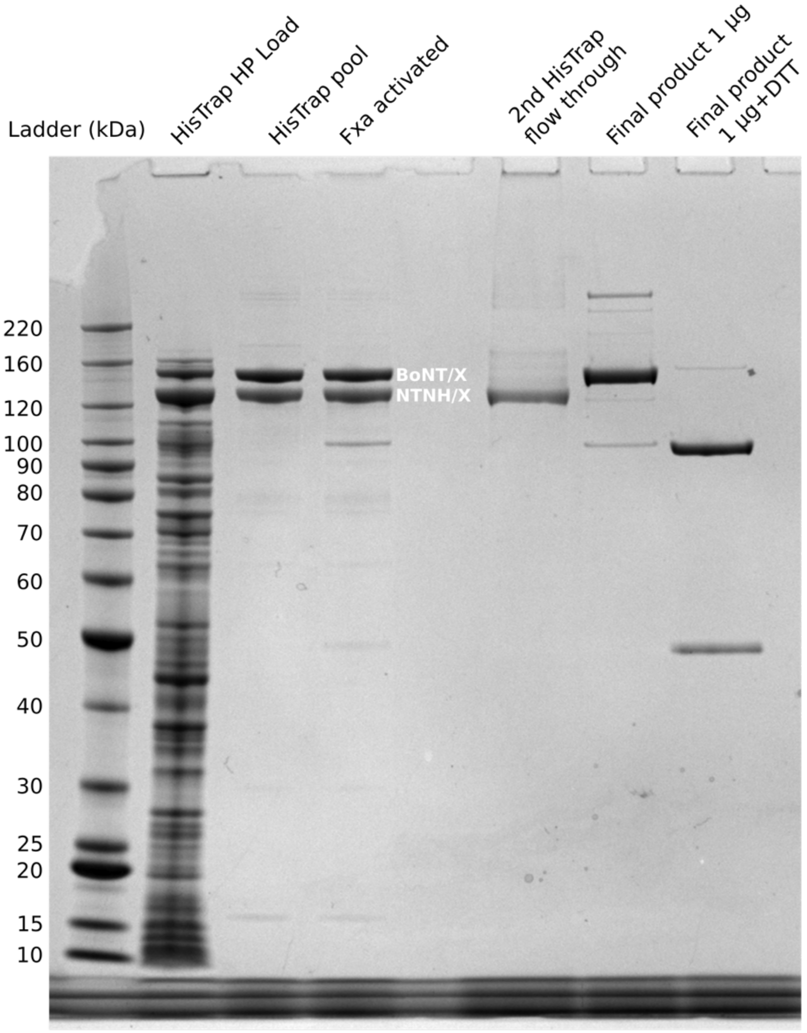

Supplementary Figure S1. Purification of BoNT/X. The lanes are marked at the top and the position of NTNH/X and BoNT/X are indicated in white. Molecular weight is indicated in kDa.

Supplementary Figure S2

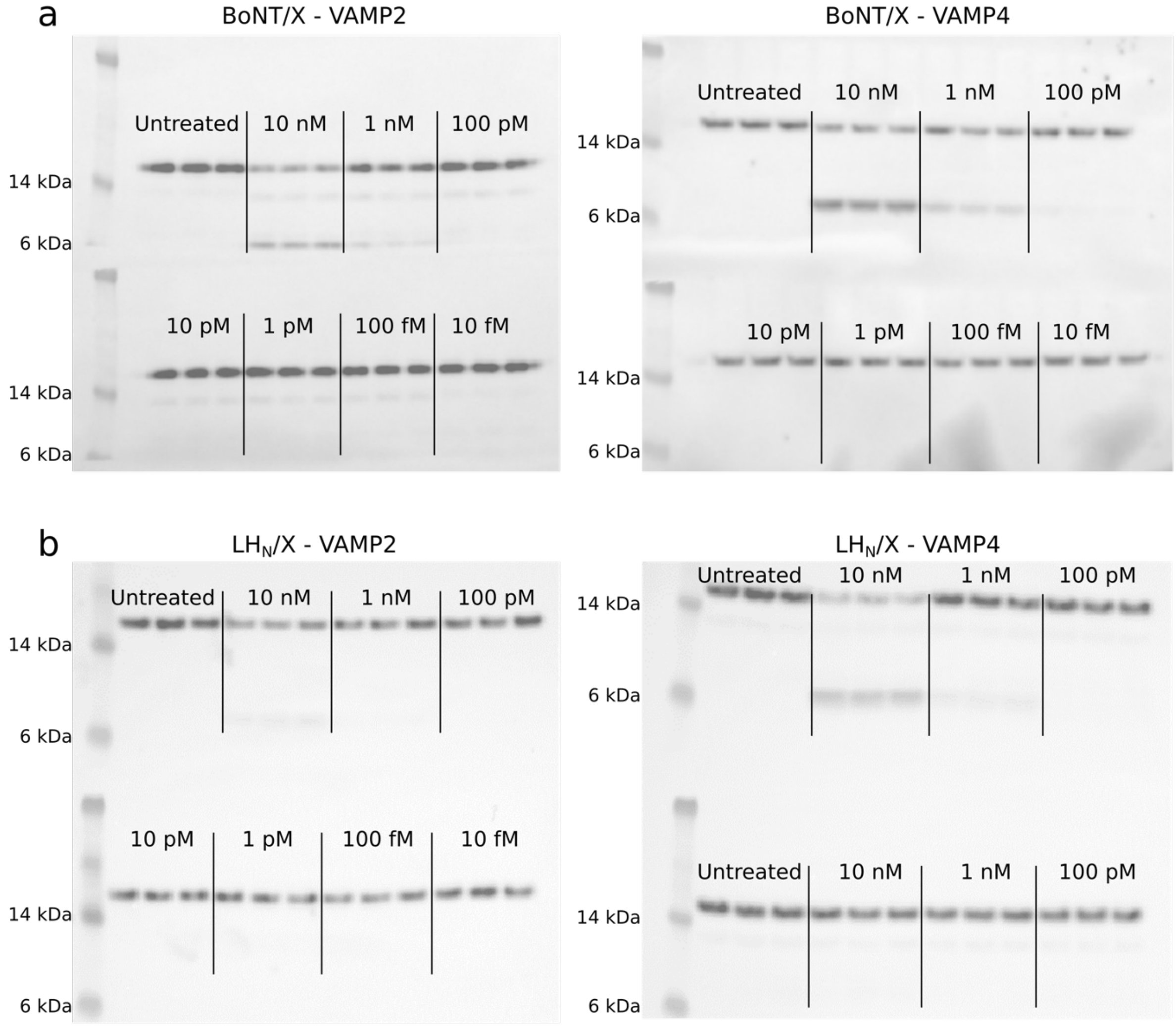

Supplementary Figure S2. Activity of BoNT/X and LH$_N$/X in cortical neurons. Rat cortical neurons were exposed to varying concentrations of recombinant full-length BoNT/X (A) or LH$_N$/X (B). After 24 h of incubation cells were lysed, and lysates analyzed for VAMP2 and VAMP4 by Western blots. VAMP migrates at about 15 kDa, whereas the VAMP cleavage product migrates at 7 kDa.

Supplementary Figure S3

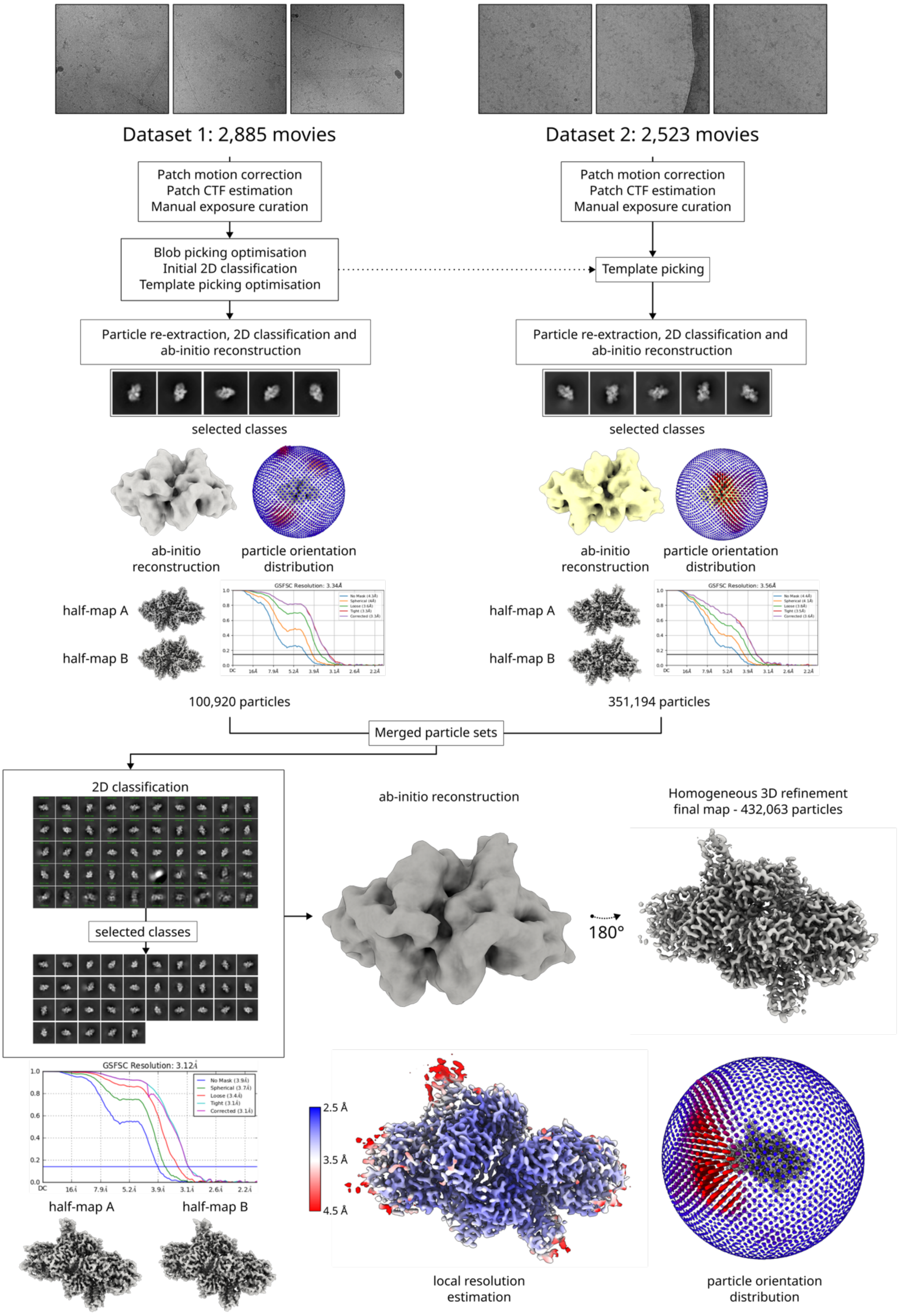

Supplementary Figure S3: cryoEM reconstruction of the BoNT/X-NTNH/X complex. Representative micrographs are shown from dataset 1 and dataset 2, and the picking strategy is outlined for each dataset in a vertical manner. Reconstructed maps, half maps, FSC curves and Euler angle distribution plots are shown for each particle set, as well as for the final map reconstruction of the merged particle sets, together with a local resolution estimation map.

Supplementary figure S4

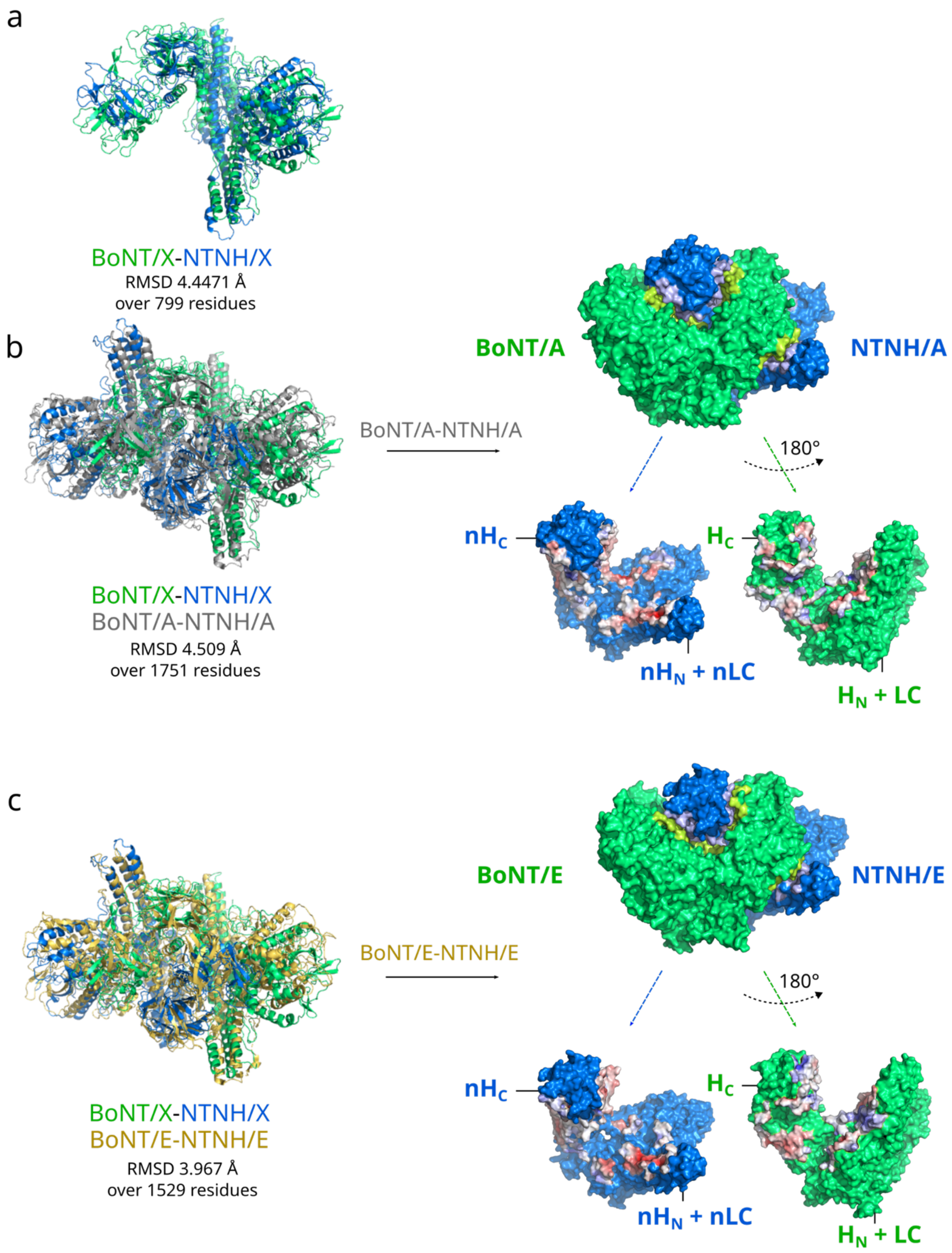

Supplementary Figure S4. (**a**) superposition of BoNT/X (green) and NTNH/X (blue), RMSD calculated over 810 Cα pairs. (**b**) superposition of BoNT/X-NTNH/X and BoNT/A-NTNH/A (PDB ID 3V0A, shown in grey). Electrostatic potential at the interface of the complex is highlighted in red (negative) and blue (positive). (**c**) superposition of BoNT/X-NTNH/X and BoNT/E-NTNH/E (PDB ID 4ZKT, shown in yellow). Acidic and basic residues on the interface are highlighted in red and blue respectively.

Supplementary figure S5

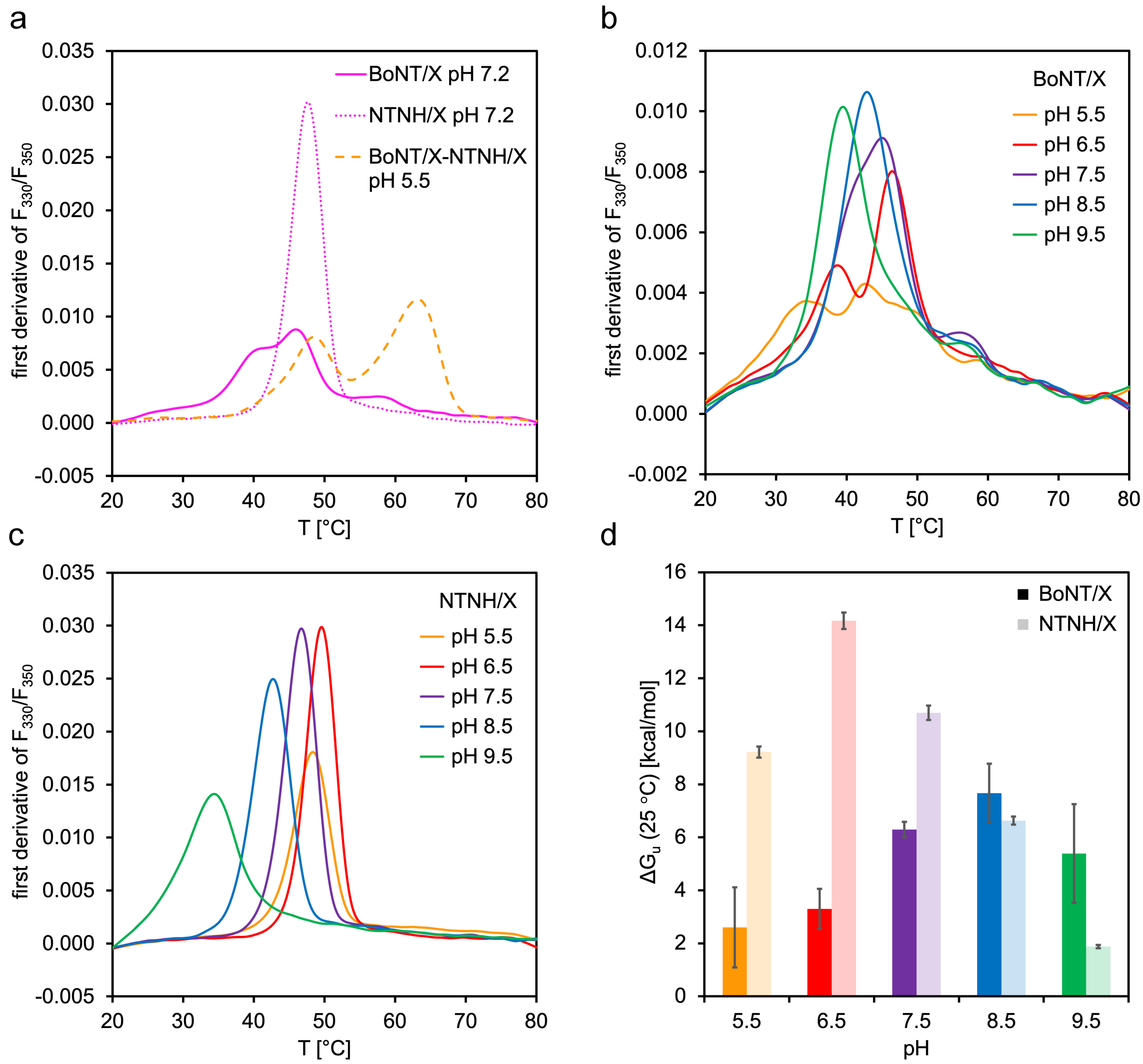

Supplementary Figure S5. (**a**) A comparison of first derivatives of nanoDSF melting curves for BoNT/X and NTNH/X at physiological neutral pH and for BoNT/X-NTNH/X at pH 5.5, at which the complex is stable. The first peak in the BoNT/X-NTNH/X curve corresponds to free NTNH/X that could not be separated from the complex in the purification by size-exclusion chromatography. A comparison of first derivatives of nanoDSF melting curves for BoNT/X and NTNH/X at various pH values are shown in panels (**b**) and (**c**), respectively. There were only minimal differences between the three individual curve replicates for each sample, and only one curve is therefore shown for each sample. (**d**) A plot of mean values and standard deviations of Gibbs free energy of unfolding (ΔG_u) at 25 °C calculated from three measured replicates of nanoDSF curves for BoNT/X and NTNH/X at various pH values. Higher ΔG_u values correspond with higher protein stability.

Supplementary figure S6

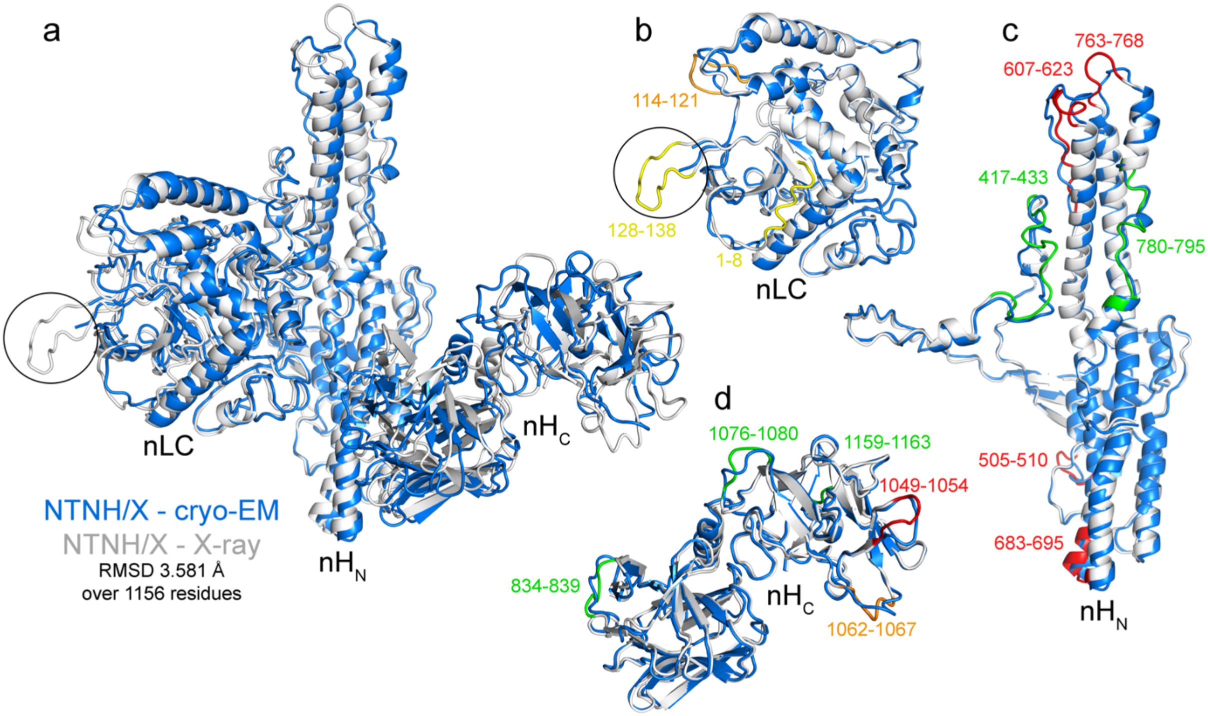

Supplementary Figure S6. (**a**) X-ray structure of free NTNH/X (grey) is shown superposed with the toxin-bound cryo-EM structure of NTNH/X (blue), demonstrating the almost identical conformation of the protein in the free and toxin-bound state, differing only slightly in the angle between nLC-nH$_N$ and nH$_C$. The panels on the right show the superposition of individual domains, nLC (**b**), nH$_N$ (**c**) and nH$_C$ (**d**). Loop regions differing in structure are highlighted in colours in the X-ray structure: green – regions involved in the toxin – NTNH interface, red – regions involved in crystal contacts, orange – regions not involved in any intermolecular contacts, and yellow – regions absent in the cryo-EM model. The extended loop (residues 128 – 139), potentially involved in interactions with other BoNT/X cluster proteins, is circled.

Supplementary Figure S7

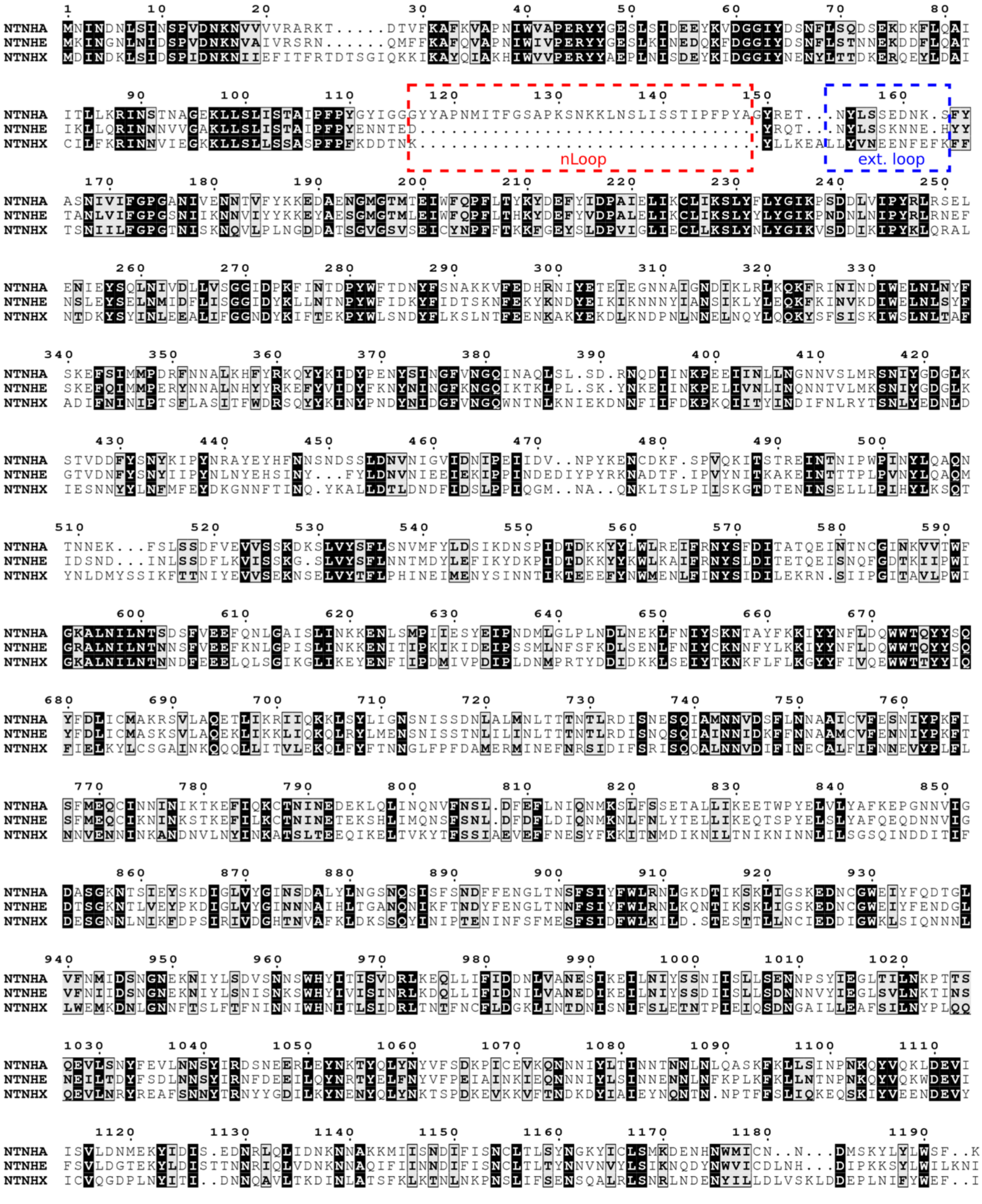

Supplementary Figure S7. Multiple sequence alignment of NTNH/A (UniProt Q45914), NTNH/E (UniProt P46082) and NTNH/X (NCBI RefSeq WP_045538950). The nLoop (residues 116 – 148) in NTNH/A is highlighted in a red box and the extended loop in NTNH/X with exposed hydrophobic residues, structurally close the NTNH/A nLoop (residues 128 – 139), is highlighted in a blue box.

Supplementary Figure S8

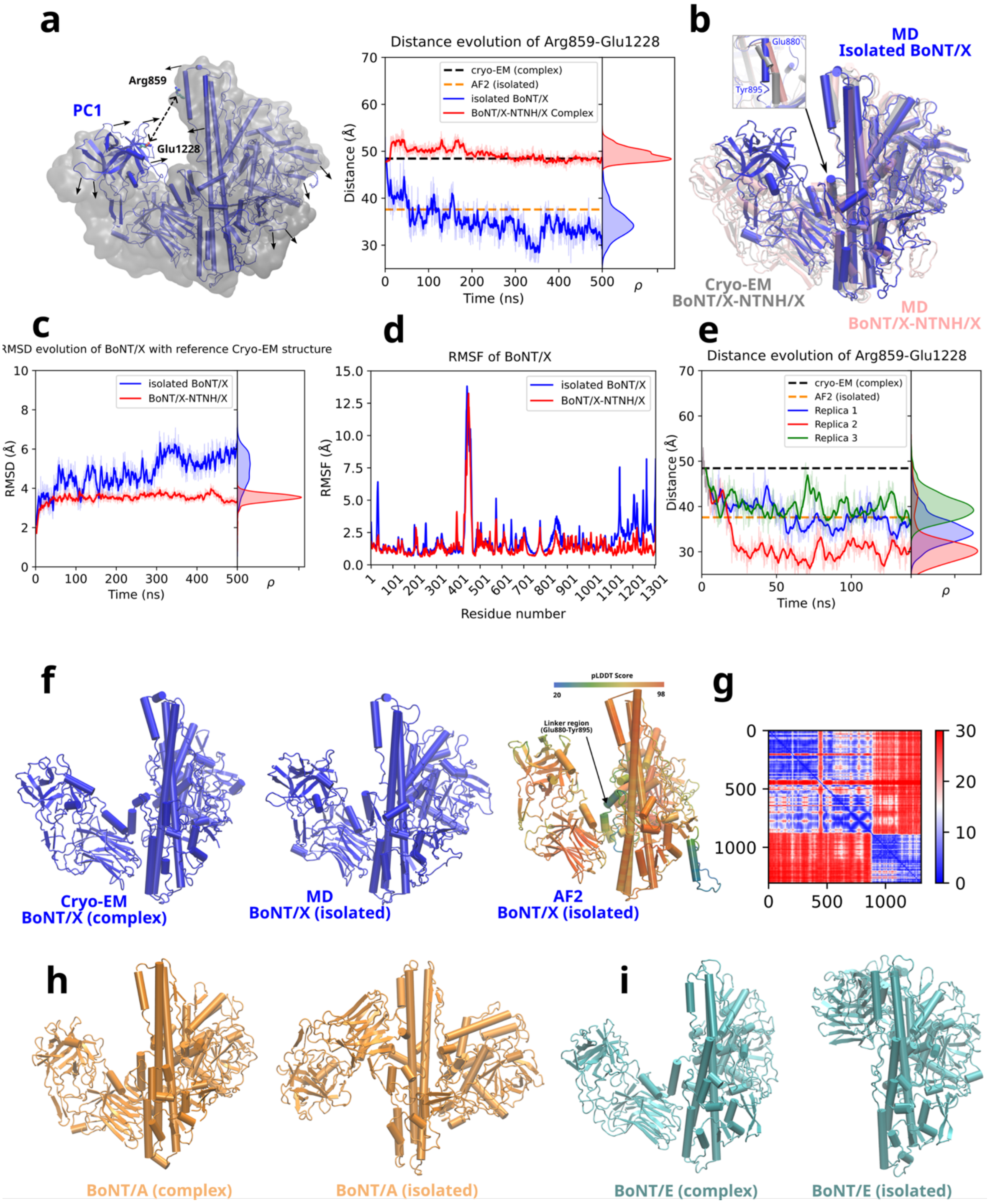

Supplementary Figure S8. Conformational dynamics of BoNT/X explored by MD simulations. (**A**) Overlay of the BoNT/X subunit (grey surface) from the cryo-EM structure of the BoNT/X-NTNH/X complex and a snapshot from the MD simulations on the isolated BoNT/X (blue cartoon). Black arrows show the motion along the main normal mode from principal component analysis of the MD simulations. Dynamics of the Arg859-Glu1228 distance from the MD simulations of isolated BoNT/X and BoNT/X-NTNH/X complex (distance from the cryo-EM structure and AlphaFold2 prediction for the isolated BoNT/X is shown with black and orange dashed line, respectively). (**B**) Overlay of BoNT/X structure from cryo-EM (transparent grey) and MD simulations of the BoNT/X-NTNH/X complex

(transparent pink) and the isolated BoNT/X subunit (blue). *Inset*: Structural changes in the linker region of the H_C domain (Glu880 to Tyr895) for the isolated BoNT/X. (**C**) Root-mean-square-deviation (RMSD) of BoNT/X for the isolated BoNT/X and the complex BoNT/X-NTNH/X structure from MD simulations. The cryo-EM structure of complex BoNT/X-NTNH/X is used as reference structure in the analysis. (**D**) Root-mean-square-fluctuations (RMSF) of BoNT/X obtained from the isolated BoNT/X and the BoNT/X-NTNH/X complex from MD simulations. The highly dynamic region in the isolated BoNT/X comprises residues 1101-1306 of the H_C domain. (**E**) Dynamics of the Arg859-Glu1228 from the three independent replicas of the MD simulations of the isolated BoNT/X. (**F**) Comparison of BoNT/X structure in the complex (cryo-EM current work, PDB ID: 8BYP) and its isolated forms (based on MD results and AlphaFold2 prediction). The AlphaFold2 prediction is colored based on the pLDDT score. The linker region (Glu880 to Tyr895) with an overall low pLDDT score is highlighted, and supports the conformational dynamics of the hinge region. (**G**) The predicted aligned error (PAE) from AlphaFold2 prediction for the isolated BoNT/X. (**H**) Comparison of BoNT/A structure in complex (PDB ID: 3V0A) and the isolated form (PDB ID: 3BTA). (**I**) Comparison BoNT/E structure in complex (PDB ID: 4ZKT) and its isolated forms (PDB ID: 3FFZ).

Supplementary table 1

BoNT/X dose	Mouse No.	Day 0 (DAS score/body weight)	Day 1 (DAS score/body weight)	Day 2 (DAS score/body weight)	Day 3 (DAS score/body weight)
1 μg/mouse	#1	0/20	0/21	0/21	0/21
	#2	0/20	0/22	0/22	0/21
	#3	0/19	0/20	0/19	0/20

1 μg of BoNT/X (0.1 μg/μl) in 10 μl of PBS was injected into the mice leg using the DAS assay and the mice were monitored for 3 days. The mice did not show any paralysis.

Supplementary table 2

BoNT/X dose	Mouse No.	Day 0 (DAS score/body weight)	Day 1 (DAS score/body weight)	Day 2 (DAS score/body weight)	Day 3 (DAS score/body weight)
2 μg/mouse	#1	0/22	0/23	0/23	0/23
	#2	0/29	0/29	0/29	0/29
	#3	0/28	0/29	0/29	0/29

2 μg of BoNT/X (0.08 μg/μl) in 25 μl of 0.1 M phosphate buffer (pH 6.1) with 0.2% gelatin were injected into the mice leg using the DAS assay and the mice were monitored for 3 days. The mice did not show any paralysis.

Supplementary table 3

	Mouse No.	Day 0 (body weight)	Day 1 (body weight)	Day 2 (body weight)	Day 3 (body weight)
BoNT/X	#1	23	23	23	24
	#2	22	22	22	23
	#3	22	21	22	22
	#4	23	23	24	23
Vehicle	#1	21	21	20	22
	#2	22	23	23	24
	#3	22	22	22	22

1 μg of BoNT/X was diluted in 100 μl of 0.1 M phosphate buffer (pH 6.1) with 0.2% gelatin and injected into CD-1 female mice intraperitoneally. A total of 4 mice were injected with BoNT/X and 3 mice were injected with vehicle only. The mice did not show obvious systemic symptoms or body weight loss.

Supplementary table 4

LH_N/X concentration (µg/mouse)	DAS Score (24 hr)	
	Unactivated LH_N/X (N=3)	Activated LH_N/X (N=3)
5	0	1
	0	1
	0	1
10	0	1
	0	2
	0	1
40	0	2
	0	2
	1	2

LH_N/X was injected to the right gastrocnemius muscle of mice (n=3) and compared to the untreated side. The limb developed paralysis after 24 hours and the toes showed a reduced ability to spread following a startle stimulus. LH_N/X that had not been activated with trypsin served as a control, which required a higher concentration and developed minimal paralysis. A quantification is shown of muscle digit abduction (DAS) scores 24 hours after injection, and a representative image of paralysis obtained from injection of 40 µg is shown in Figure 1e. For quantitative values, means are presented ± the standard deviation.

Supplementary table 5. Cryo-EM data collection, refinement and validation statistics.

	BoNT/X-NTNH/X (EMD-16330) (PDB 8BYP)	
**Data collection and processing**		
Dataset	1	2
Magnification	130,000×	130,000×
Voltage (kV)	300	300
Electron exposure ($e^-/Å^2$)	35.6	35.9
Defocus range (µm)	-1.8 – -3.4	-1.8 – -3.4
Pixel size (Å)	1.05	1.05
Initial particle images (no.)	591,151	1,123,595
Final particle images (no.)	432,063	
Symmetry imposed	none	
Map resolution (Å)	3.12	
FSC threshold	0.143	
Map resolution range (Å)	2.5 – 5.0	
**Refinement**		
Initial model used (PDB code)	3v0a	
Model resolution (Å)	2.9/3.3	
FSC threshold	0.143/0.5	
Map sharpening *B* factor ($Å^2$)	-95.7	
Model composition		
Non-hydrogen atoms	19,836	
Protein residues	2,421	
B factors ($Å^2$)		
Protein	64.31	
R.m.s. deviations		
Bond lengths (Å)	0.005	
Bond angles (°)	1.007	
Validation		
MolProbity score	1.93	
Clashscore	9.98	
Poor rotamers (%)	0.4	
Ramachandran plot		
Favored (%)	93.78	
Allowed (%)	6.22	
Disallowed (%)	0	

Supplementary table 6. NanoDSF assay. Mean values and standard deviations calculated from three measured replicates are listed for denaturation onset temperature, melting temperatures (T_m), and Gibbs free energy of unfolding (ΔG_u) at 25 °C. Higher ΔG_u values correspond with higher protein stability. NA – not applicable.

Sample	Onset [°C]	T_m1 [°C]	T_m2 [°C]	ΔG_u (25 °C) [kcal/mol]
BoNTX pH 7.2	31.70 ± 0.04	45.68 ± 0.09	NA	5.63 ± 0.19
NTNH/X pH 7.2	41.66 ± 0.20	47.64 ± 0.09	NA	12.28 ± 0.51
BoNTX-NTNH/X pH 5.5	40.88 ± 0.08	48.36 ± 0.08	62.94 ± 0.06	NA
BoNT/X pH 5.5	20.38 ± 0.66	33.72 ± 0.81	44.01 ± 0.06	2.60 ± 1.51
BoNT/X pH 6.5	20.65 ± 1.13	38.50 ± 0.17	46.40 ± 2.68	3.30 ± 0.76
BoNT/X pH 7.5	32.96 ± 0.20	44.27 ± 0.47	NA	6.29 ± 0.29
BoNT/X pH 8.5	34.82 ± 0.23	42.40 ± 0.45	NA	7.67 ± 1.11
BoNT/X pH 9.5	30.48 ± 2.79	38.27 ± 1.15	NA	5.39 ± 1.86
NTNH/X pH 5.5	41.52 ± 0.38	48.27 ± 0.03	NA	9.22 ± 0.21
NTNH/X pH 6.5	44.01 ± 0.06	49.53 ± 0.02	NA	14.17 ± 0.31
NTNH/X pH 7.5	40.38 ± 0.14	46.52 ± 0.14	NA	10.70 ± 0.27
NTNH/X pH 8.5	35.57 ± 0.06	42.67 ± 0.04	NA	6.63 ± 0.15
NTNH/X pH 9.5	20.00 ± 0.00	34.39 ± 0.08	NA	1.88 ± 0.06

Supplementary table 7. X-ray data collection and refinement statistics.

	NTNH/X (PDB 8QFT)
**Data collection and processing**	
Space group	$P3_121$
Cell dimensions	
a, b, c (Å)	174.11, 174.11, 138.02
α, β, γ (°)	90.00, 90.00, 120.00
Resolution (Å)	87.05-3.30 (3.45-3.30)*
R_{merge}	0.202 (2.772)
$I / \sigma I$	7.0 (0.6)
CC(1/2)	0.999 (0.924)
Completeness (%)	100 (100)
Redundancy	19.3 (16.5)
**Refinement**	
Resolution (Å)	73.74-3.30 (3.39-3.30)
No. reflections	34,592 (2,541)
R_{work} / R_{free}	0.276 / 0.333 (0.550 / 0.557)
No. atoms	
Protein	9,710
Ligand/ion	0
Water	0
B-factors	
Protein	220.05
Ligand/ion	NA
Water	NA
R.m.s. deviations	
Bond lengths (Å)	0.004
Bond angles (°)	1.044

* Values in parentheses are for highest-resolution shell.

Supplementary table 8. Rmsd values for superpositions of H_C/X with other BoNT receptor-binding domains mentioned in the structural analysis section.

**Structures**	**Rmsd (Å)**	**Cα pairs**
H_C/X – H_C/D (PDB ID 3OGG)	1.653	345
H_C/X – H_C/F (PDB ID 3FUQ)	1.515	373
H_C/X – H_C/E (PDB ID 4ZKT)	1.980	356
H_C/X – H_C/A (PDB ID 5JLV)	1.915	388
H_C/B (free, PDB ID 2NM1) – H_C/B (receptor bound, PDB ID 1Z0H)	0.566	441

Supplementary table 9. Estimation of pK_a values of residues involved in BoNT/X-NTNH/X interaction, calculated for the isolated subunits and the complex structure. The pK_a shift (ΔpK_a) resulting from BoNT/X-NTNH/X interaction is also reported, *ΔpK_a = pK_a (complex) – pK_a (isolated). Residues Glu1048, nGlu554, and nGlu584 show large pK_a shifts upon complexation. The p*K*a values were computed using electrostatic model as implemented in PropKa3.0.[86]

Residue	Chain	pK_a (isolated)	pK_a (complex)	ΔpK_a*
K637	X	10.5	10.2	-0.3
D639	X	4.3	5.8	1.6
K867	X	10.2	9.8	-0.4
D998	X	4.2	6.0	1.8
K1000	X	10.6	9.8	-0.7
K1046	X	11.2	12.3	1.1
E1048	X	3.7	7.6	3.9
E1049	X	4.1	4.5	0.4
K1050	X	11.0	12.6	1.6
D1051	X	3.9	4.6	0.7
K1013	X	10.8	10.6	-0.2
D1018	X	3.9	4.4	0.5
D1128	X	3.3	4.7	1.4
K1129	X	10.6	14.6	4.1
R1185	X	13.0	13.6	0.6
R1232	X	11.9	12.5	0.6
K1235	X	10.1	9.4	-0.7
E1282	X	4.4	5.8	1.5
R1287	X	12.4	12.3	-0.1
K1291	X	10.0	12.3	2.3
E99	N	5.3	6.5	1.2
K255	N	10.4	10.9	0.5
D317	N	4.6	5.0	0.4
D432	N	4.1	3.1	-1.0
K428	N	10.4	9.8	-0.6
E554	N	4.5	7.1	2.6
R556	N	13.0	14.6	1.7
E584	N	6.1	7.4	1.3
K636	N	10.5	11.8	1.3
E787	N	2.9	2.9	0.1
E789	N	4.8	4.8	0.0
E793	N	4.8	5.7	0.9
E833	N	4.9	3.8	-1.1
E920	N	4.9	4.6	-0.3
E1013	N	4.6	5.1	0.4
E1084	N	3.8	4.8	1.0
