# Supplementary material for: Activity of botulinum neurotoxin X and its structure when shielded by a non-toxic non-hemagglutinin protein
Source: Commun Chem. 2024 Aug 13;7:179. doi: 10.1038/s42004-024-01262-8 (PMC11322297; doi:10.1038/s42004-024-01262-8)
Supplement: Supplementary file 3 — Description of Additional Supplementary Files [file 42004_2024_1262_MOESM3_ESM.pdf]

Description of Additional Supplementary Files

File name- Supplementary Movie 1.

File description- Principal component analysis of the isolated BoNT/X subunit, showing large-scale conformational changes in molecular dynamics simulations.

File name- Supplementary Movie 2.

File description- Principal component analysis of the BoNT/X-NTNH/X complex, showing conformational changes during the molecular dynamics simulations. The BoNT/X and NTNH/X subunits are depicted in blue and red, respectively, while the NTNH/X subunits are shown in transparent.

File name- Supplementary Data 1.

File description- Raw data of the ganglioside binding assays.
