# Supplementary material for: Activity of botulinum neurotoxin X and its structure when shielded by a non-toxic non-hemagglutinin protein
Source: Commun Chem. 2024 Aug 13;7:179. doi: 10.1038/s42004-024-01262-8 (PMC11322297; doi:10.1038/s42004-024-01262-8)
Supplement: Supplementary file 7 — Reporting Summary [file 42004_2024_1262_MOESM7_ESM.pdf]

nature portfolio

Corresponding author(s): Min Dong and Pål Stenmark

Last updated by author(s): Jun 5, 2024

# Reporting Summary

Nature Portfolio wishes to improve the reproducibility of the work that we publish. This form provides structure for consistency and transparency in reporting. For further information on Nature Portfolio policies, see our Editorial Policies and the Editorial Policy Checklist.

## Statistics

For all statistical analyses, confirm that the following items are present in the figure legend, table legend, main text, or Methods section.

| n/a | Confirmed | |
|---|---|---|
| ☐ | ☒ | The exact sample size (*n*) for each experimental group/condition, given as a discrete number and unit of measurement |
| ☐ | ☒ | A statement on whether measurements were taken from distinct samples or whether the same sample was measured repeatedly |
| ☒ | ☐ | The statistical test(s) used AND whether they are one- or two-sided<br>*Only common tests should be described solely by name; describe more complex techniques in the Methods section.* |
| ☒ | ☐ | A description of all covariates tested |
| ☒ | ☐ | A description of any assumptions or corrections, such as tests of normality and adjustment for multiple comparisons |
| ☒ | ☐ | A full description of the statistical parameters including central tendency (e.g. means) or other basic estimates (e.g. regression coefficient) AND variation (e.g. standard deviation) or associated estimates of uncertainty (e.g. confidence intervals) |
| ☒ | ☐ | For null hypothesis testing, the test statistic (e.g. *F*, *t*, *r*) with confidence intervals, effect sizes, degrees of freedom and *P* value noted<br>*Give P values as exact values whenever suitable.* |
| ☒ | ☐ | For Bayesian analysis, information on the choice of priors and Markov chain Monte Carlo settings |
| ☒ | ☐ | For hierarchical and complex designs, identification of the appropriate level for tests and full reporting of outcomes |
| ☒ | ☐ | Estimates of effect sizes (e.g. Cohen's *d*, Pearson's *r*), indicating how they were calculated |

*Our web collection on statistics for biologists contains articles on many of the points above.*

## Software and code

Policy information about availability of computer code

| | |
|---|---|
| Data collection | EPU 2.7 (Thermo Fischer); UNICORN 5.2 control and analysis software (Cytiva); DNASTAR Lasergene 12; IOX software (Emka technologies); Syngene GeneGnome (Syngene Bioimaging, Cambridge UK). |
| Data analysis | cryoSPARC 3.1; Coot 0.8.5.0; Phenix 1.19; UCSF Chimera version 1.13.1; PyMOL Molecular Graphics System version 2.2.3; gromacs v2019.5; GraphPad Prism 7; SEC: UNICORN 5.2 control and analysis software (Cytiva).<br>GeneTools software (Syngene Bioimaging, Cambridge, UK); CCP4Interface 8.0.; Coot 0.9.8.7.; Phenix version 1.20.1-4487. |

For manuscripts utilizing custom algorithms or software that are central to the research but not yet described in published literature, software must be made available to editors and reviewers. We strongly encourage code deposition in a community repository (e.g. GitHub). See the Nature Portfolio guidelines for submitting code & software for further information.

## Data

Policy information about availability of data

All manuscripts must include a data availability statement. This statement should provide the following information, where applicable:

- Accession codes, unique identifiers, or web links for publicly available datasets
- A description of any restrictions on data availability
- For clinical datasets or third party data, please ensure that the statement adheres to our policy

Three-dimensional cryo-EM maps generated during this study have been deposited in the Electron Microscopy Data Bank (EMDB) under accession code

EMD-16330. Coordinates of the model have been deposited in the Protein Data Bank (PDB) under accession code 8BYP.
The model coordinates and the electron density maps for the crystal structure of NTNH/X
were deposited into the PDB database under the accession code 8QFT.

## Human research participants

Policy information about studies involving human research participants and Sex and Gender in Research.

| | |
|---|---|
| Reporting on sex and gender | *Use the terms sex (biological attribute) and gender (shaped by social and cultural circumstances) carefully in order to avoid confusing both terms. Indicate if findings apply to only one sex or gender; describe whether sex and gender were considered in study design whether sex and/or gender was determined based on self-reporting or assigned and methods used. Provide in the source data disaggregated sex and gender data where this information has been collected, and consent has been obtained for sharing of individual-level data; provide overall numbers in this Reporting Summary. Please state if this information has not been collected. Report sex- and gender-based analyses where performed, justify reasons for lack of sex- and gender-based analysis.* |
| Population characteristics | *Describe the covariate-relevant population characteristics of the human research participants (e.g. age, genotypic information, past and current diagnosis and treatment categories). If you filled out the behavioural & social sciences study design questions and have nothing to add here, write "See above."* |
| Recruitment | *Describe how participants were recruited. Outline any potential self-selection bias or other biases that may be present and how these are likely to impact results.* |
| Ethics oversight | *Identify the organization(s) that approved the study protocol.* |

Note that full information on the approval of the study protocol must also be provided in the manuscript.

# Field-specific reporting

Please select the one below that is the best fit for your research. If you are not sure, read the appropriate sections before making your selection.

☒ Life sciences ☐ Behavioural & social sciences ☐ Ecological, evolutionary & environmental sciences

For a reference copy of the document with all sections, see nature.com/documents/nr-reporting-summary-flat.pdf

# Life sciences study design

All studies must disclose on these points even when the disclosure is negative.

| | |
|---|---|
| Sample size | In the first cryo-EM data set, 2,885 movies were recorded, from which 70 were rejected. A total of 591,151 particles were automatically picked in cryoSPARC, and after 2D classification 100,920 particles were selected for 3D refinement. In the second dataset, 1,123,595 particles were automatically picked from 2,523 recorded movies, and 351,194 particles were selected in 2D classification and used for 3D refinement. Finally, the particle sets from both datasets were combined for 2D classification and subsequent 3D refinement, which included 432,063 particles and yielded the final map at 3.12 Å resolution, calculated based on the gold-standard FSC of 0.143.<br><br>The size-exclusion chromatography was performed in a single experiment. |
| Data exclusions | All movies were analyzed and 70 movies were excluded from further analysis due to low quality and ice artifacts.<br><br>No data were excluded in the size-exclusion chromatography experiment. |
| Replication | All cryo-EM replication attempts (protein preparation, cryo-EM grid preparation, data collection and analysis) were successful. The cryo-EM data were collected in a total of 2 independent data collection sessions.<br><br>The size-exclusion chromatography was performed with a single sample for each pH value.<br><br>The proteolysis assay was performed more than 2 times independently with same outcome.<br><br>Standard deviations were calculated based on ≥3 biological replicates. The information is given in Materials and Methods section and in Extended figures legends.<br><br>VAMP cleavage data (mean + S.E.M) were calculated from three independent experiments performed in triplicate.<br>Mouse PNHD data (mean ± S.E.M ) were calculated from three independent experiments per concentration |
| Randomization | No randomization was relevant for single particle cryo-EM study. Randomization is not relevant for this type of structural biology work. The collected cryo-EM data are random by nature.<br><br>No randomization is applicable to the size-exclusion chromatography experiment. |

| | |
|---|---|
| Blinding | The cryo-EM image processing workflow includes systematic blinding steps, such as lowpass filtering of particle picking templates in order to avoid introducing bias in the picking models. Additionally, the resolution of the map is assessed via the spatial correlation of two half maps, each of them independently reconstructed from a random half-set of particles.<br><br>No blinding is applicable to the size-exclusion chromatography experiment. |

# Reporting for specific materials, systems and methods

We require information from authors about some types of materials, experimental systems and methods used in many studies. Here, indicate whether each material, system or method listed is relevant to your study. If you are not sure if a list item applies to your research, read the appropriate section before selecting a response.

## Materials & experimental systems

| n/a | Involved in the study |
|---|---|
| ☐ | ☒ Antibodies |
| ☐ | ☒ Eukaryotic cell lines |
| ☒ | ☐ Palaeontology and archaeology |
| ☐ | ☒ Animals and other organisms |
| ☒ | ☐ Clinical data |
| ☒ | ☐ Dual use research of concern |

## Methods

| n/a | Involved in the study |
|---|---|
| ☒ | ☐ ChIP-seq |
| ☒ | ☐ Flow cytometry |
| ☒ | ☐ MRI-based neuroimaging |

## Antibodies

| | |
|---|---|
| Antibodies used | Invitrogen 6x-His Tag Monoclonal Antibody (AD1.1.10), HRP ((ThermoFisher #MA1-80218)<br>monoclonal rabbit VAMP-2 (custom made by Abcam), monoclonal mouse VAMP4 (Santa Cruz sc-365332), HRP-conjugated ant-rabbit IgG (Sigma A6154), HRP-conjugated anti-mouse IgG (Sigma A4416). |
| Validation | Mouse anti Histidine tag antibody, clone AD1.1.10, recognizes proteins and peptides containing the motif HHHHHH<br>VAMP4 recognises sequence DEVIDVMQENITKVIERGERLDELQDKSESLSDNATAFSNRSKQLRRQMWWRGCKIKAIMALVAA. |

## Eukaryotic cell lines

Policy information about cell lines and Sex and Gender in Research

| | |
|---|---|
| Cell line source(s) | Primary cortical neurons were prepared from Sprague Dawley rats (CD®) embryos (embryonic day 17-18). |
| Authentication | *Describe the authentication procedures for each cell line used OR declare that none of the cell lines used were authenticated.* |
| Mycoplasma contamination | Primary cells were not tested for mycoplasma. |
| Commonly misidentified lines<br>(See ICLAC register) | *Name any commonly misidentified cell lines used in the study and provide a rationale for their use.* |

## Animals and other research organisms

Policy information about studies involving animals; ARRIVE guidelines recommended for reporting animal research, and Sex and Gender in Research

| | |
|---|---|
| Laboratory animals | Hsd:ICR (CD-1) mice, Envigo, (6-8 weeks of age, 17 – 20 g bodyweight)<br>Housing conditions: dark/light cycle (12 h), ambient temperature (25 degree) and humidity (30%-40%).<br>Hemi-diaphragms were obtained from adult male, CD-1 mice (25-30 g bodyweight) purchased from Charles River (Margate, UK).<br>Cortical neurons were prepared from Sprague Dawley rats (CD®) purchased from Charles River rat embryos (embryonic day 17-18). |
| Wild animals | No wild animals were used in this study |
| Reporting on sex | DAS assay was done in the female mice. Hemi-diaphragm tissue was obtained from male mice. |
| Field-collected samples | No field collected samples were used in this study. |
| Ethics oversight | All animal studies, including euthanasia via carbon dioxide asphyxiation, were conducted according to ethical regulations under protocols approved by the Institute Animal Care and Use Committee (IACUC) at Boston Children's Hospital (00001465). |

Note that full information on the approval of the study protocol must also be provided in the manuscript.
